# Knowledge and attitudes of female genital mutilation among midwives in Eastern Sudan

**DOI:** 10.1186/1742-4755-9-23

**Published:** 2012-09-28

**Authors:** Abdel Aziem A Ali

**Affiliations:** 1 Department of Obstetrics and Gynecology, Faculty of Medicine, Kassala University, P.O. Box 496, Kassala, Sudan

**Keywords:** Female, Genital mutilation, Midwives, Sudan

## Abstract

**Background:**

Female Genital Mutilation (FGM) or cutting caries legal and bioethical debates and it is practiced in many developing countries.

**Methods:**

Random selection of 154 midwives was used for the study during June 2012 and through July 2012 aiming to assess knowledge and attitudes of the midwives towards FGM in Eastern Sudan.

**Results:**

A total of 157 midwives enrolled in this study. They had been practicing for 3 – 44 years (mean SD 19.2 ± 10.3). More than two third of them experienced practicing FGM sometime in their life (127/157, 80.9%). There was low level of awareness of types of FGM practice since only 7% (11/157) identified the four types correctly. 53.5% (84/157) identified type 1 correctly while 18.5% (29/157), 17.8% (28/157) and 15.9% (25/157) identified type 2, 3 and 4 as correct respectively. While 30 (19.1%) of the midwives claimed that all types of FGM are harmful, 76.4% (120/157) were of the opinion that some forms are not harmful and 7 (4.5%) reported that all types of FGM are not harmful. Likewise while 74.5% (117/157) of the interviewed midwives mentioned that the FGM is a legal practice only 25.5% (40/117) were of the opinion that FGM is illegal practice. The vast majority of the respondents (64.3%, 101/157) have an opinion that FGM decreases the sexual pleasure. More than half (53.5%, 84/157) of the participants affirmed that FGM does not increase the risk of HIV transmission. High proportion of the respondents (71.3%, 112/157) did not know whether or not infertility could complicate FGM.

**Conclusions:**

Thus a substantial effort should be made to discourage the continuation of FGM practice among midwives in Sudan. This might be achieved by improving knowledge and awareness among the midwives and the community

## Background

Female Genital Mutilation (FGM) or cutting caries legal and bioethical debates and it is practiced in 28 African countries and some Asian countries [1]. World Health Organization defined FGM as ‘’all procedures involving partial or total removal of the external female genitalia or other injury to the female organs whether for cultural or other non-therapeutic reasons [2]. WHO and other United Nations Organizations classified FGM into four types: type1, also known as clitoridectomy or *Suna*: involves partial or total removal of the clitoris and/or prepuce; type2: involves partial or total removal of the clitoris and labia minora, with or without excision of the labia majora; type3: also known as infibulation or *pharaonic*, it entails removing part or all of the external genitalia and narrowing the vaginal orifice by re-approximating the labia minora and/or labia majora; type4: includes any form of other harm done to the female genitalia by pricking, piercing, cutting, scraping or burning [3].

Girls typically undergo the procedures between the age of 6 and 12 years and the cutting is always performed by the midwives without anesthesia, antibiotics or even sterilization, putting the girls in short and long term very serious consequences [4]. In Sudan there is a very high prevalence rate of FGM (ranged between 87%-100%) [5]. Pharaonic type is the most prevalent type in some area of Sudan including Kassala State. Female genital mutilation in Sudan is performed irrespective of females’ social or religious groups and the law in the Sudan forbids the practice of FGM. In the present there is no governmental or social attempt to eliminate the practice and the uncircumcised girl looks odd and unmarriageable [5,6]. Thus the current study is directed to assess knowledge and attitudes of the midwives towards FGM and it is expected to provide the health planners with fundamental data for the development of strategies that might reduce this practice.

## Methods

### Participants

Kassala State, Eastern Sudan is 42282 square kilometer, populated by 1.8 million and it is nearly 600 kilometer from Khartoum, capital of Sudan with a prominent diversity in culture, religion, language and ethnicity. Random selection of 154 midwives was used for the study during June 2012 and through July 2012. Midwives used in this study were traditional birth attendants and they were identified by local community members. After informed consent these midwives answered face to face interview at their home using an opened questionnaire. Ten medical students were selected as interviewers, they had been trained in relationship building, local cultures, believes, privacy, confidentiality and how to administer the questionnaires. Information sought in the questionnaire included socio-demographic characteristics (age, residence, education, duration of experience), knowledge about types of FGM, whether it is harmful or not, whether it is legal or not, whether they routinely practice it, how many girls they circumcised in the last year, whether they will perform FGM in the future and are they going to expose their daughter to FGM or not?, those who practiced FGM were asked about the reasons lying behind their performing FGM. The respondents were also asked to highlight the complications of FGM and whether FGM increases the incidence of HIV infection and infertility or not.

### Data analysis

Data were entered into a computer database and SPSS software (SPSS Inc., Chicago, IL, USA, version 13.0) and double checked before analysis. Means and proportions for the socio-demographic characteristics were compared between the circumciser and non-circumciser midwives using student and *x*^2^ test, respectively and *P* < 0.05 was considered significant.

### Ethics

The study received ethical clearance from the Research Board at Ministry of Health Kassala State, Eastern Sudan.

## Results

### Midwives characteristics

The age of 157 midwives participating in the study ranged between 24 – 65 year (mean SD 43.9 ± 8.8) and most of them (99/157, 63.1%) were illiterate. They had been practicing for 3 – 44 years (mean SD 19.2 ± 10.3). More than two third of them experienced practicing FGM sometime in their life (127/157, 80.9%) and in the last year preceded the study the range of the practice was reported as 5 – 88 times among these 127 midwives.

### Awareness and knowledge of FGM among midwives

Among the respondents there was low level of awareness of types of FGM practice since only 7% (11/157) identified the four types correctly. 53.5% (84/157) identified type 1 correctly while 18.5% (29/157), 17.8% (28/157) and 15.9% (25/157) identified type 2, 3 and 4 as correct respectively. While 30 (19.1%) of the midwives claimed that all types of FGM are harmful, 76.4% (120/157) were of the opinion that some forms are not harmful and 7 (4.5%) reported that all types of FGM are not harmful. Likewise while 74.5% (117/157) of the interviewed midwives mentioned that the FGM is a legal practice only 25.5% (40/117) were of the opinion that FGM is illegal practice. The vast majority of the respondents (64.3%, 101/157) have an opinion that FGM decreases the sexual pleasure. More than half (53.5%, 84/157) of the participants affirmed that FGM does not increase the risk of HIV transmission. High proportion of the respondents (71.3%, 112/157) did not know whether or not infertility could complicate FGM.

### Identified complications and attitudes

Of the possible complications hemorrhage was identified by the majority of the respondents (84.7%, 133/157) followed by infection and pain (33 and 44 respectively). Nearly two thirds (66.2%, 104/157) of the respondents stated that they will practice FGM in the future and 37% (58/157) would not have their daughters circumcised. The reasons for non-willing to practice FGM in the future mentioned by 53 respondents were illegal and the complications of FGM.

When we compared the educational status (illiteracy) between the respondents who intended and did not intend to continue the practice (104 Vs 53 participants respectively) there was no significant statistical difference between the two groups (67\104 (64.4%) Vs 32\53 (60.4%), *P* value = 0.5). Likewise the educational status was not varied between those who were aware of all types of FGM and those who were not (7\11, 63.6% Vs 95/146, 65.1%, *P* value = 0.9).

### Reasons for practicing FGM and comparison between the circumciser and non-circumciser midwives

Reasons given by the respondents for practicing FGM were cultural (51.2%, 65/127), religion (26%, 33/127) and economic (22.8%, 29/157). In comparison between the circumciser and non-circumciser midwives there was no significant statistical difference in socio-demographic data (age, duration of experience and residence) and knowledge concerning identification of all types of FGM, Table 1.

**Table 1:** Comparison between the circumciser and non-circumciser midwives in Eastern Sudan, 2012

**Variable**	**circumciser (N = 127)**	**Non-circumciser (N = 30)**	**P**
Age, years	43.8 (8.9)	44.3 (8.7)	0.7
Duration of work, years	19.3 (10)	18.7 (11.3)	0.7
Illiteracy	80 (63%)	19 (63.3)	0.5
Rural residence	58 (45.7%)	14 (46.7%)	0.5
Identified all types of FGM correctly	8 (6.3%)	3 (10%)	0.3

Data was shown in mean (SD) and number (%).

## Discussion

The finding of this study showed that there was low level of awareness concerning the different types of FGM among the midwives in Eastern Sudan. Moreover the majority were of the opinion that some forms are not harmful and nearly 55 of the respondents stated that they will practice FGM in the future. FGM is recognized as a violation of human and child rights and outlawed in many countries [1]. Mediclizing FGM on ethical ground is strongly opposed by the international medical community [7]. Eradication of FGM practice necessitates a substantial effort to improve knowledge and awareness among the community. Grassroots programmes organized by the international bodies that focus on improving the human’s right awareness and knowledge have had great success in reducing the incidence of the practice. Sudan is one of African countries with very high prevalence rate of female genital cutting, though the findings of this study indicate that without addressing the midwives any strategy to reduce the practice ultimately could fail. In Sudan there is lack of support by religion and law to fight the FGM practice and we believe that the confusing role of the religion and ambiguous law are essential reasons for the continuation of the FGM practice. Thus involvement of religion persons and educationalists together with a clear cut law to punish the circumcisers will decrease its prevalence in this country [6].

In this study despite 80.9% of the respondents practiced FGM there is paucity of knowledge in the classification of FGM. This was in agreement with study in Alexandria where 6.7% of the respondent’s nurses identified the three types correctly [8]. While 76.4% of the midwives felt that some forms of FGM are not harmful, only 19.1% of the respondents stated that all types of FGM are a harmful practice. Moreover 66.2% claimed that they will continue to practice FGM. It is not surprising to note this contradiction among our respondents; this is because strong social reasons maintain the high level of FGM among Sudanese girls. In most areas in Sudan uncircumcised girl is viewed as odd and unmarriageable [5], this strongly influences the midwives to continue in practicing FGM. The vast majority of the respondents have an opinion that FGM decreases the sexual pleasure, however Okonofua et al. reported that FGM did not attenuate sexual feeling and that it may predispose women to adverse sexuality outcomes such as early pregnancy and genital tract infection [9]. The scar of the mutilated genitalia leads to painful coitus and thus adverse effect concerning the sexual feeling. In Sub-Saharan Africa there is increase in HIV infection [10] and of course FGM will increase its spread by non-sterilization thus it is a real public problem to know that more than half of the participants affirmed that FGM practice does not constitute a risk factor for HIV transmission. It is not yet known whether infertility complicates FGM or not thus it is not unexpected that the majority of the respondents did not known if infertility does not complicate FGM [11]. FGM is a public health issue with recognized complications, hemorrhage was reported by the majority of the respondents and this goes with what is reported among Nigerian nurses [12]; however it is much higher than in Alexandria study where 20.7% mentioned hemorrhage as a complication [8].

## Conclusions

In conclusion our findings showed that almost all the midwives in our community are practicing FGM, they have very low level of awareness regarding the different types of the FGM. The majority of the respondents did not view the practice as harmful and insisted to continue in practicing it for cultural reason. Thus a substantial effort should be made to discourage the continuation of the practice among midwives in Sudan. This might be achieved by improving knowledge and awareness among the midwives and the community.

## Competing interests

We declare that we have no conflict of interest.

## Author’s contributions

The author alone is responsible for the content and writing of the paper.
